# The prediction of Alzheimer’s disease through multi-trait genetic modeling

**DOI:** 10.3389/fnagi.2023.1168638

**Published:** 2023-07-27

**Authors:** Kaylyn Clark, Wei Fu, Chia-Lun Liu, Pei-Chuan Ho, Hui Wang, Wan-Ping Lee, Shin-Yi Chou, Li-San Wang, Jung-Ying Tzeng

**Affiliations:** ^1^Department of Pathology and Laboratory Medicine, Penn Neurodegeneration Genomics Center, Perelman School of Medicine, University of Pennsylvania, Philadelphia, PA, United States; ^2^Department of Pathology and Laboratory Medicine, Perelman School of Medicine, University of Pennsylvania, Philadelphia, PA, United States; ^3^Department of Health Management and Systems Sciences, School of Public Health and Information Sciences, University of Louisville, Louisville, KY, United States; ^4^Leonard Davis Institute of Health Economics, University of Pennsylvania, Philadelphia, PA, United States; ^5^Department of Economics, Lehigh University, Bethlehem, PA, United States; ^6^National Bureau of Economic Research, Cambridge, MA, United States; ^7^Department of Statistics, North Carolina State University, Raleigh, NC, United States; ^8^Bioinformatics Research Center, North Carolina State University, Raleigh, NC, United States

**Keywords:** Alzheimer’s disease, statistical genetics, genetic risk, risk scores, polygenic trait

## Abstract

To better capture the polygenic architecture of Alzheimer’s disease (AD), we developed a joint genetic score, MetaGRS. We incorporated genetic variants for AD and 24 other traits from two independent cohorts, NACC (*n* = 3,174, training set) and UPitt (*n* = 2,053, validation set). One standard deviation increase in the MetaGRS is associated with about 57% increase in the AD risk [hazard ratio (HR) = 1.577, *p* = 7.17 E-56], showing little difference from the HR for AD GRS alone (HR = 1.579, *p* = 1.20E-56), suggesting similar utility of both models. We also conducted APOE-stratified analyses to assess the role of the e4 allele on risk prediction. Similar to that of the combined model, our stratified results did not show a considerable improvement of the MetaGRS. Our study showed that the prediction power of the MetaGRS significantly outperformed that of the reference model without any genetic information, but was effectively equivalent to the prediction power of the AD GRS.

## 1. Introduction

### 1.1. Genetic risk scores

A genetic risk score (GRS), also known as a polygenic risk score, is an estimate of an individual’s genetic risk for a trait of interest. To calculate a simple GRS, the weighted sum of an individual’s single nucleotide polymorphism (SNP) genotypes in the target dataset is computed; the SNPs involved can be from the entire genome or some pre-determined genomic locations, and the weights are the SNP effect sizes, typically obtained from a publicly available, large scale genome-wide association study (GWAS) that are referred to as the base dataset. The simplest way to calculate a risk score is the pruning/clumping and thresholding (P+T or C+T) method, which selects SNPs for inclusion that pass linkage disequilibrium (LD) pruning/clumping and *p*-value thresholding (Clark et al., 2022). A GRS can be used to stratify individuals based on trait-specific genetic risk, to conduct trait prediction in an independent dataset, and to study shared genetic basis among different traits.

### 1.2. Previous development of a stroke MetaGRS

Abraham et al. (2019) developed MetaGRS for ischemic stroke (IS). Some previous studies (Ibrahim-Verbaas et al., 2014; Malik et al., 2014) had indicated the limited predictive power of ischemic stroke GRS, while others (Inouye et al., 2018; Maier et al., 2018) had shown that GRS can be made more powerful when using summary statistics from GWAS for multiple phenotypes. To leverage this information, Abraham et al. (2019) calculated GRS for 19 different traits, including IS, and incorporated them into an ischemic stroke MetaGRS. They found that the inclusion of risk information for multiple types of stroke and stroke-related phenotypes led to a slightly better prediction power than the IS GRS alone. The superiority of MetaGRS models, though marginal, has also been shown for other traits, including type 2 diabetes and coronary artery disease (Inouye et al., 2018; Wünnemann et al., 2019; Chen et al., 2021).

### 1.3. Motivation to develop an Alzheimer’s disease MetaGRS

Alzheimer’s disease (AD) has been previously shown to have a polygenic architecture (Escott-Price et al., 2015; Leonenko et al., 2021), with Escott-Price et al. (2015) indicating that an AD polygenic risk score had increased predictive ability when compared to a conventional logistic regression model including only APOE genotype and relevant covariates. However, the variance explained (R^2^) by these risk score models only reaches about 0.29 whereas Alzheimer’s disease has a heritability of up to 80% (Gatz et al., 2006; Leonenko et al., 2021; Bellenguez et al., 2022). Because of this discrepancy, we aimed to construct MetaGRS following the procedure described by Abraham et al. (2019) in an effort to produce a more powerful AD risk score model. The implementation of this model will allow us to explore a more effective tool to capture genetic risk at an earlier stage, leading to the possibility of earlier interventions for AD.

## 2. Results

### 2.1. Derivation of MetaGRS for AD

**FIGURE 1:**
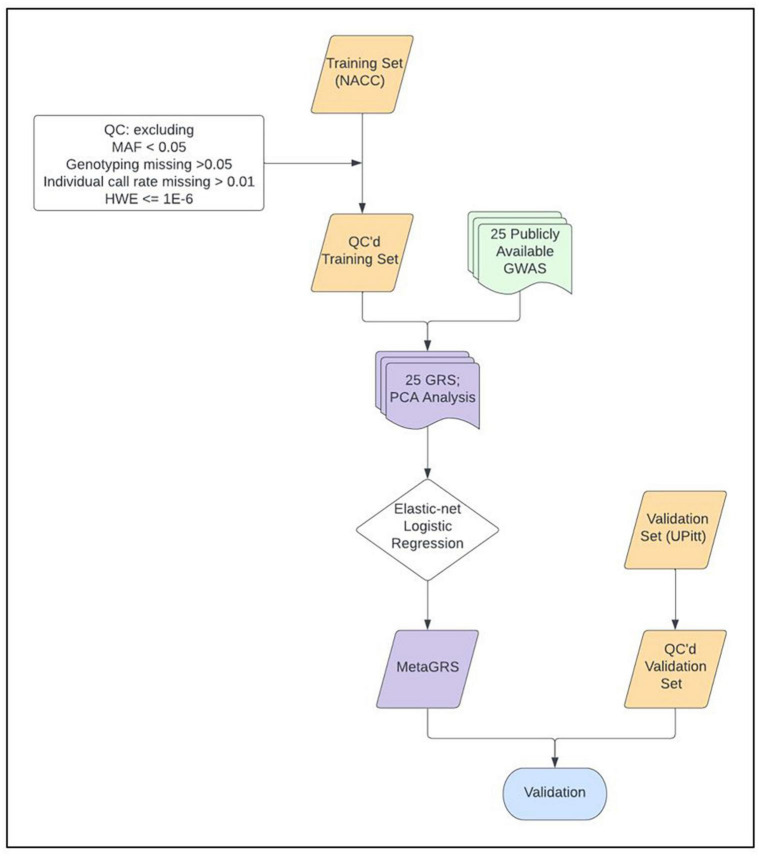
MetaGRS workflow. The workflow we followed to derive our Alzheimer’s disease MetaGRS. The MetaGRS model was trained using the quality controlled (QC’ed) NACC genotype data and the GWAS summary statistics files for 25 phenotypes as listed in Supplementary Table 1. The constructed MetaGRS model is then applied to the UPitt dataset to evaluate its predictive power.

**FIGURE 2:**
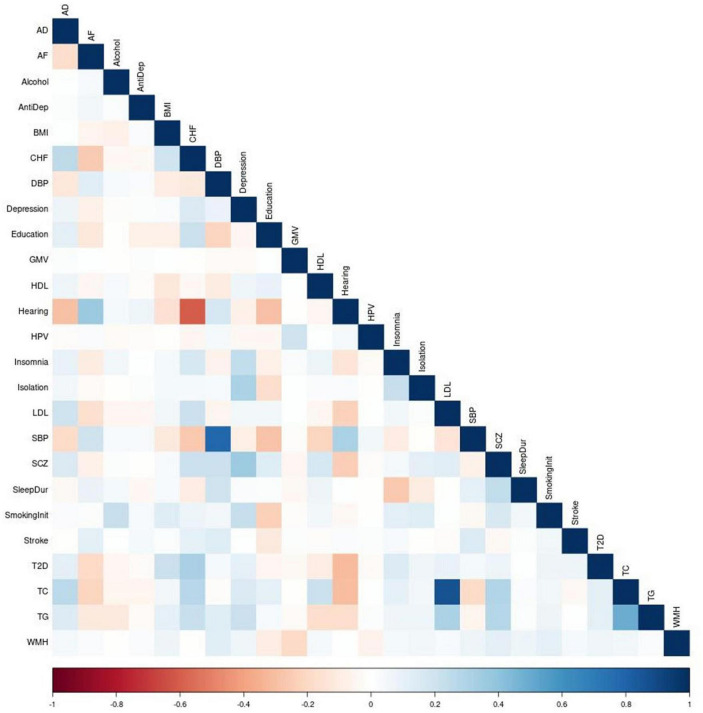
Correction matrix of PCA-GRSs. Pairwise correlations between the PCA-GRSs of each phenotype. Dark red indicates a strong negative correlation while dark blue indicates a strong positive correlation.

We construct and train the MetaGRS for AD using the samples from the National Alzheimer’s Coordinating Center (NACC; *n* = 3,174, Table 1); we evaluate its predictive power in an independent test dataset, i.e., the Alzheimer’s Disease Research Center (ADRC) samples housed at the University of Pennsylvania (ADRC-UPitt), which is the largest non-NACC dataset in the Alzheimer’s Disease Genetics Consortium (ADGC) and consists of 2,053 individuals (Table 1). The workflow is presented in Figure 1. We obtain GWAS summary statistics for AD and 24 other phenotypes (Ripke et al., 2013; Willer et al., 2013; Locke et al., 2015; Christophersen et al., 2017; Day et al., 2018; Elliott et al., 2018; Evangelou et al., 2018; Lee et al., 2018; Malik et al., 2018; Xue et al., 2018; Dashti et al., 2019; Howard et al., 2019; Jansen et al., 2019; Kunkle et al., 2019; Liu et al., 2019; Wells et al., 2019; Persyn et al., 2020; Shah et al., 2020; Wigmore et al., 2020) (see Supplementary Table 1 for phenotype names, abbreviations, and sources) to construct GRS for each phenotype in NACC. All GRSs, including the AD GRS, are constructed using SNPs from all autosomes. We use the P+T approach and adopt 13 *p*-value thresholds (see “Materials and methods” section). Because some phenotype data are unavailable in NACC, we do not use phenotype data to select the optimal *p*-value threshold as in the original P+T method. Instead, for each phenotype, we follow Coombes et al. (2020) and perform principal component analysis (PCA) on the 13 GRSs, each obtained from a *p*-value threshold. We then retain the first principal component for use in the rest of the analysis (hereafter, PCA-GRS). Figure 2 shows the correlation among the PCA-GRS of different phenotypes — DBP, Hearing, and SBP are among the phenotypes that are negatively associated with AD, while CHF, LDL, SCZ, TC, and TG display positive associations.

**TABLE 1:** Data summary.

	NACC				UPitt			
	All	Control	AD	*p*-value^2^	All	Control	AD	*p*-value^2^
Characteristic	*N* = 3,174^1^	*N* = 1,512^1^	*N* = 1,662^1^		*N* = 2,049^1^	*N* = 810^1^	*N* = 1,239^1^	
AD	1,662 (52%)				1,239 (60%)			
Sex	1,298 (41%)	510 (34%)	788 (47%)	<0.001	759 (37%)	299 (37%)	460 (37%)	>0.9
**Apoe type**								
e2e2	9 (0.3%)	8 (0.5%)	1 (<0.1%)	0.017	8 (0.4%)	5 (0.6%)	3 (0.2%)	0.3
e2e3	239 (7.5%)	175 (12%)	64 (3.9%)	<0.001	152 (7.4%)	111 (14%)	41 (3.3%)	<0.001
e3e3	1,499 (47%)	924 (61%)	575 (35%)	<0.001	1,009 (49%)	534 (66%)	475 (38%)	<0.001
e3e4	1,093 (34%)	349 (23%)	744 (45%)	<0.001	693 (34%)	134 (17%)	559 (45%)	<0.001
e4e4	253 (8.0%)	25 (1.7%)	228 (14%)	<0.001	133 (6.5%)	9 (1.1%)	124 (10%)	<0.001
e2e4	81 (2.6%)	31 (2.1%)	50 (3.0%)	0.087	54 (2.6%)	17 (2.1%)	37 (3.0%)	0.2

^1^n (%); Median (IQR).

^2^ Fisher’s exact test.

Detailed breakdown of the post-QC’ed NACC and UPitt datasets. Indicates the disease status and sex breakdown, along with the distribution of APOE genotype among samples. *P*-values were obtained using Fisher’s exact test comparing AD case and control groups.

Of note, some PCA-GRSs are correlated (for example, SBP and DBP, CHF and Hearing in Figure 2), and therefore contribute overlapping information. We use regularization to account for the correlations when constructing the MetaGRS. We used elastic net regression with AD status as the dependent variable and the 25 PCA-GRS as predictors to determine the regression coefficients of each PCA-GRS, adjusting for sex and the first 5 principal components. The formula for MetaGRS is shown in the “Materials and methods” section. We use 20-fold cross validation in NACC data, and the model that maximizes the area under the receiver-operating characteristic curve (AUC) us chosen as the final model.

**FIGURE 3:**
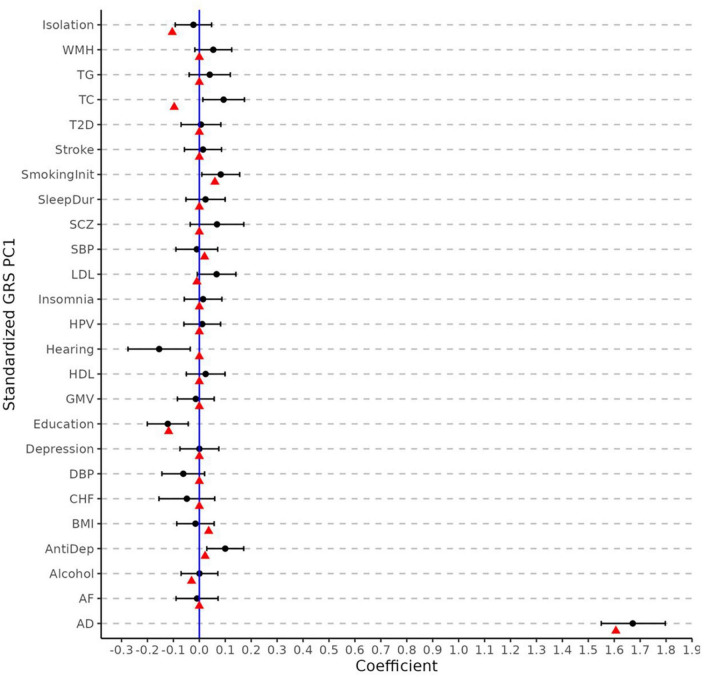
Elastic net regression coefficients and univariate logistic regression. The weights (i.e., log odd ratio) for each phenotype from elastic net regression (red points) and from the univariate logistic regression of each phenotype (black points) with its 95% confidence intervals. The coefficients are obtained using standardized PCA-GRS which have zero mean and unit standard deviation for each phenotype. All regression analyses adjust for sex and the first five principal components.

Figure 3 displays the regression coefficients from elastic net regression (hereafter referred to as MetaGRS weights). As comparison, we also plot the coefficients and the associated 95% confidence interval (CI) from logistic regressions that use AD status as the dependent variable and the corresponding phenotype’s PCA-GRS as the predictor, adjusting for the same covariates as in the elastic-net model. After accounting for the effects and correlations across different phenotypes, the majority of the non-AD phenotypes contribute a null MetaGRS weight. Specifically, AD, which has the largest absolute weight in the single PCA-GRS logistic regression, still contributes to the largest weight in the MetaGRS, (1.606) and dominates other phenotypes. In contrast, several other phenotypes (e.g., LDL, Hearing, and Antidepressant Use) have non-trivial regression coefficients in the single PCA-GRS logistic regression but contribute negligibly in the MetaGRS. In the MetaGRS Smoking Initiation contributes the second largest positive weight, while Isolation and Education contribute the largest negative weights.

### 2.2. Evaluating MetaGRS

**FIGURE 4:**
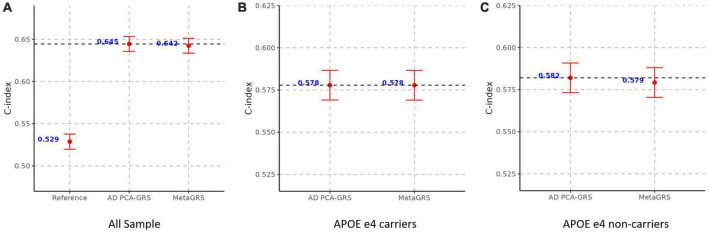
MetaGRS validation using Cox proportional hazard model in UPitt. The figure shows the performance of MetaGRS in UPitt in **(A)** all sample; **(B)** APOE e4 carriers; and **(C)** APOE e4 non-carriers. Cox proportional hazard model is performed in each sample, adjusting for age, sex and the first five principal components. C-index is used to evaluate the performance of prediction.

We construct the MetaGRS for AD in the UPitt testing data (*n* = 2,053) and evaluate its performance using both Cox proportional hazard model and logistic regression. The MetaGRS shows a significant improvement in the C-index compared to the reference model which includes sex and the first five principal components (C-index for MetaGRS = 0.642, C-index for the reference model = 0.529, Figure 4A). Similar patterns were seen in Abraham et al. (2019) and Chen et al. (2021). Nevertheless the C-index suggests the MetaGRS has a predictive power equivalent to AD PCA-GRS (C-index for MetaGRS = 0.642, C-index for AD PCA-GRS = 0.645, Figure 4A). This is also indicated by the hazard ratios (HR) – one standard deviation increase in the MetaGRS is associated with about 57% increase in the AD risk [hazard ratio (HR) = 1.577, *p* = 7.17 E-56, Table 2], showing a minimal difference from the HR for AD PCA-GRS alone (HR = 1.579, *p* = 1.20E-56, Table 2). This minimal improvement of MetaGRS is not unique to AD. The predictive power of MetaGRS for T2D is not distinguishably better than the T2D GRS (Chen et al., 2021) alone; the MetaGRS for stroke has a slightly better power than the IS GRS alone, with a magnitude of less than 0.02 improvement in C-index (Abraham et al., 2019).

**TABLE 2:** MetaGRS validation in UPitt – Cox hazard model.

Sample	N	PGS	Beta	HR	S.E.	P
All sample	2052	AD PCA-GRS	0.457	1.579	0.029	1.20E-56
		Meta PCA-GRS	0.456	1.577	0.029	7.17E-56
APOE e4 carrier	880	AD PCA-GRS	0.210	1.233	0.046	4.86E-06
		Meta PCA-GRS	0.209	1.232	0.046	6.17E-06
APOE e4 non-carrier	1169	AD PCA-GRS	0.288	1.334	0.056	2.89E-07
		Meta PCA-GRS	0.283	1.327	0.055	3.24E-07

Cox hazard model results for the AD PCA-GRS and the Meta PCA-GRS. Results include those for the full UPitt validation sample and stratified into APOE e3 carriers and non-carriers. HR, hazard ratio; S.E., standard error. The sample size of all sample is 2052, among of which 2049 has non-missing information about the APOE status, and 3 with missing information about the APOE status.

To confirm that our finding is not sensitive to the model specification, we also evaluate the MetaGRS by using logistic regression that takes AD status (a binary variable) as the dependent variable and adjust for age, sex, and the first 5 principal components. When using logistic regressions, we use AUC to compare the prediction performance. The AUC for MetaGRS is 0.7110, effectively the same as the AUC of 0.7113 for AD PCA-GRS (Supplementary Table 2). The receiver operating characteristic (ROC) curve for MetaGRS is also indistinguishable from the curve for AD PCA-GRS (Supplementary Figure 1), showing very little improvement in the prediction power in MetaGRS. Previous studies have identified the dominant predictive power of the APOE e4 allele (Stocker et al., 2021), thus motivating us to evaluate the influence of APOE status on the MetaGRS.

### 2.3. Stratification analysis by APOE e4 status

We further evaluate the predictive power of MetaGRS in samples split according to APOE e4 status. Not surprisingly, due to the e4 allele’s well-known status as the main genetic driver of AD, we do not observe any improvement of the MetaGRS in APOE e4 carriers based on the Cox hazard model estimates (C-index = 0.578 for AD PCA-GRS, C-index = 0.578 for MetaGRS in Figure 4B, HR = 1.233 for AD PCA-GRS, HR = 1.232 for MetaGRS in Table 2). Among the APOE e4 non-carriers, the MetaGRS does not render a considerable improvement in the prediction power compared to AD PCA-GRS (Figure 4C and Table 2). Validations using logistic regression again confirm the minimal superiority of MetaGRS. The only difference in the results from logistic regression is, among the APOE e4 non-carriers, the odds ratio (OR) for MetaGRS is slightly higher than the OR for AD PCA-GRS, as are the AUC and pseudo R^2^.

### 2.4. Adding APOE e2 and e4 dosage as covariates

As a sensitivity analysis, we additionally include APOE e2 and e4 dosage as covariates into the elastic net model in the training stage and the Cox model in the validation stage. Compared to Figure 4A, we see that adding APOE dosage covariates significantly improves the performance of the reference model, AD PCA-GRS model, and MetaGRS model (Supplementary Figure 2A). Unlike the considerable difference in the C-index between the reference model and MetaGRS in Figure 4A, the gap is narrowed, which echoes the dominant role of APOE region in AD risk prediction. Despite these changes, the MetaGRS does not exhibit a better performance of risk prediction against the AD PCA-GRS (Supplementary Figure 2), i.e., MetaGRS and AD PCA-GRS have near identical C-index values in all samples and in e4-stratified samples.

### 2.5. Removing APOE region in calculation of AD GRS

**FIGURE 5:**
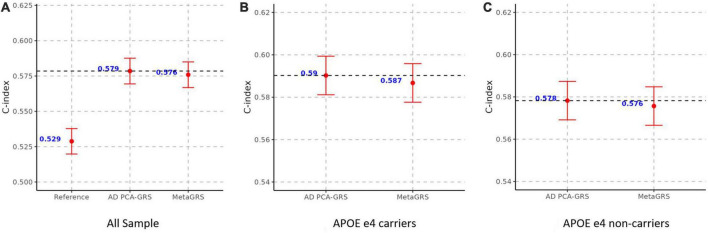
MetaGRS validation using Cox proportional hazard model in UPitt after removing APOE region. The figure shows the performance of MetaGRS in UPitt in **(A)** all sample; **(B)** APOE e4 carriers; and **(C)** APOE e4 non-carriers. APOE region is removed in the calculation of AD GRS. Cox proportional hazard model is performed in each sample, adjusting for age, sex and the first five principal components. C-index is used to evaluate the performance of prediction.

Lastly, we construct an additional AD-GRS by removing the APOE region and adjusting for age, sex, and the first 5 principal components, and replicated all the rest of the training and validation process. Removing the APOE region leads to a slightly smaller weight of AD PCA-GRS in the elastic net regression in the training stage (weight = 1.499 in Supplementary Figure 3 compared to weight = 1.606 in Figure 2), while Isolation an Education remain the two phenotypes with the largest negative weights (Supplementary Figure 3). There are significant changes in the magnitude of predictive risk – one standard deviation increase in the MetaGRS is associated with about 26% increase in the AD risk (HR = 1.259, *p* = 1.45E-15, Supplementary Table 3), compared to an increase of 57% when APOE region is included in the calculation of AD GRS (HR = 1.577, *p* = 7.17E-15, Table 2). This is not surprising considering we excluded the region with the most hazardous genetic variants for AD. Though MetaGRS still outperforms the reference model, there is little evidence supporting its superiority over AD PCA-GRS which is again manifested in all individuals, individuals with APOE e4 alleles, including very similar values in C-index (Figure 5), HR (Supplementary Table 3), and OR, pseudo-R^2^, and AUC (Supplementary Table 4) between MetaGRS and AD PCA-GRS.

## 3. Discussion

Our study showed that MetaGRS for AD significantly outperformed the reference model that includes no genetic covariates, but was almost equivalent to AD GRS in terms of the prediction power. Our results do not stand against current literature on Stroke (Abraham et al., 2019) and T2D (Chen et al., 2021)−the MetaGRS does show considerable improvement in prediction power when evaluated against the predictive performance of the GRS of single risk factors, however, the improvement is negligible or nonexistent when benchmarked against the GRS of the phenotype of interest.

We explored possible reasons behind the limited superiority of MetaGRS. Figure 3 shows that the AD GRS overwhelms the contributions of other phenotypes in MetaGRS (e.g., Isolation, Education). As shown in Figure 3, both AD MetaGRS weight and AD logistic regression coefficient are significantly different from zero while other phenotypes have their MetaGRS weights and logistic regression coefficients either near zero or substantially smaller than AD. By nature of the polygenic structure of AD, one possible cause could be the dominant role of APOE variants in AD genetic risk. We investigated the impact of APOE further with additional analyses−stratification analysis by APOE e4 status, including APOE e2 and e4 dosage as covariates when constructing the AD GRS, and removing the APOE region in the calculation of AD GRS. All of these analysis results speak to the fact that APOE variants are not the primary cause of the minimal improvement from MetaGRS.

We are aware that many other important explanations are not explored in this paper, all of which could be possible avenues for future exploration. First, most MetaGRS studies, including ours, focus on European ancestry only, warranting investigation of the power of MetaGRS in other ancestries. For example, APOE e4 allele frequency varies across ethnicities, [e.g., 37% (14%) for AD cases (controls) in Caucasian, 32% (19%) for AD cases (controls) in African Americans, 19% (11%) for AD cases (controls) in Hispanic, and 28% (9%) for AD cases (controls) in Japanese] (Farrer et al., 1997). In AD studies, the hazard ratio of APOE e4/e4 also shows discrepancy across ancestries [OR is 12.5, 5.7, 2.2, and 33.1 in Caucasians, African Americans, Hispanics, and Japanese, respectively (Farrer et al., 1997)]. It is worth investigating whether MetaGRS performs better for ethnicities with a smaller APOE e4 frequency in the future.

Secondly, MetaGRS did not consider environmental and behavioral risk factors and their interactions with genetic factors, thus, social determinants of health were overlooked. Literature has shown an association between neighborhood disadvantage and AD (Powell et al., 2020), where living in a more disadvantaged neighborhood is associated with a higher risk of AD. Consistent access to healthcare resources, for which we did not control for, benefits both physical and brain health and may explain a non-negligible portion of variations for diseases related to AD or AD itself (Livingston et al., 2020) and should be further studied. Lastly, there may be important but undiscovered risk variants for AD that we could not include into the MetaGRS model thus the modest improvement of the MetaGRS for AD. We expect this to be addressed as larger, more diverse genome-wide association (GWA) studies are performed and results released to the public.

The results presented here suggest that the AD MetaGRS is effectively as clinically useful as the typical AD GRS, though both can stand to be improved. AD MetaGRS can help to classify individuals into different groups based on their AD genetic risk. This is crucial for AD as the current treatment is primarily focused on symptom management. For individuals with a high AD genetic risk, preventative interventions should be taken earlier to slow down the disease progression. In clinical trials, AD MetaGRS can be an alternative proxy to assist with selection of the highest risk subjects in order to improve the likelihood of finding effective prevention therapies (Clark et al., 2022).

There could be several avenues for future research. First, the biological explanations underlying the similarities in prediction power between AD GRS and MetaGRS are not fully explained in this study. Further studies could explore the functional interpretation of the genetic variants included in the MetaGRS and their potential biological relevance to AD. Moreover, our study is focused on European White subjects. As larger, more diverse GWA studies are performed, we expect more studies to replicate MetaGRS in other ethnicities.

## 4. Materials and methods

### 4.1. Study design and participants

#### 4.1.1. Training data–NACC samples

The National Alzheimer’s Coordinating Center (NACC) is responsible for maintaining a database of clinical information collected from the 29 NIA-funded Alzheimer’s Disease Centers (ADCs) (Beekly et al., 2004). Each center collects and manages patient information in a site-specific way, requiring data harmonization on the part of NACC. To train our MetaGRS model we created a combined dataset from ADCs 1-7, which included genetic and AD diagnosis information for 5,869 subjects (2,494 AD cases, 2,021 controls, and 1,354 missing/unknown) before QC.

#### 4.1.2. Testing data−ADRC University of Pittsburgh (UPitt) samples

While NACC oversees the data from the ADCs, is also falls under a group of Alzheimer’s Disease Research Centers (ADRCs). These 33 ADRCs are NIA-funded medical centers aimed at translational AD research (Alzheimer’s Disease Research Centers, 2021) spread across 26 states. For our testing dataset we used the ADRC housed at the University of Pittsburgh, the largest non-NACC ADGC dataset. This input dataset contained 2,212 subjects, with a 60/40 case-control split, before QC.

#### 4.1.3. Identifying phenotypes of interest

As mentioned previously, GRS calculation requires GWAS summary statistics, meaning our MetaGRS requires summary statistics for multiple traits. In order to pick the most informative phenotypes for inclusion in our MetaGRS model, we conducted a literature search of all NACC studies, with no inclusion restrictions, to identify risk factors and comorbidities correlated with AD. From our initial list of more than 50 traits, we excluded those without a publicly available large-scale GWAS. We then cross-referenced our list with those included in Andrews et al. (2021), a study that investigated individual trait risk scores and their relationship with AD. From this, we narrowed our list to 25 traits for inclusion, including AD, BMI, type 2 diabetes, and depression (see Supplementary Table 1 for a full list). For each phenotype, we use the GWAS summary statistics based on European White ancestry and built upon the Human Genome Build 19 or GRCh 37.

#### 4.1.4. Data cleaning and quality control

Both the training and testing datasets were originally separated into individual chromosomes in genfile format. In order to easily work with the data, we converted all files into PLINK’s bfile format and then merged all chromosomes into a single whole-genome file. We then removed any samples of non-European heritage and proceeded to the quality control (QC) process as described in Choi et al. (2020), with small changes to parameters to fit our needs, using PLINK 1.90beta version 6.9 and R. We performed the following QC procedures on the NACC and UPitt datasets: (1) removing SNPs with a minor allele frequency < 0.05, significant (*p* < 1e-6) Hardy-Weinberg equilibrium test values, and missing in more than 5% of subjects; (2) removing samples missing more than 1% of genotyped SNPs; (3) removing samples with extreme heterozygosity estimate values; (4) removing mismatching SNPs between the GWAS and training/testing data and correcting SNPs that needed to be recoded, strand flipped, or both; (5) removing individuals with a first or second degree relative in the sample, as indicated by a relatedness value greater than 0.125.

All 25 GWAS summary statistics files were QC’ed the same way, again following the procedures laid out in Choi et al. (2020). GWAS QC involved removing SNPs with a minor allele frequency < 0.01, SNPs that were duplicates or indels, and ambiguous SNPs.

### 4.2. GRS calculation for each phenotype

The first step to calculate MetaGRS is to calculate GRS for each phenotype. Because not all 25 phenotypes are available in NACC samples, we adopt the principal component approach of Coombes et al. (2020) to calculate PCA-GRS of each phenotype. Specifically, given a phenotype *k*, we compute GRS of subject *i* using *GRS*_*ik*(*t*)_ = ∑_*j*_ β_*jk*_*x*_*ij*_*I* {*p*_*jk*_ < *t*} for a *p*-value threshold *t*, where β_*jk*_ is the effect size for SNP *j* from the GWAS summary statistics for phenotype *k*, *x*_*ij*_ is the minor allele count of SNP *j* for subject *i*, and *p*_*jk*_ is the *p*-value of SNP *j* for phenotype *k*. We consider 13 *p*-value thresholds for *t*: 5e-8, 1e-7, 1e-6, 1e-5, 1e-4, 1e-3, 0.01, 0.05, 0.1, 0.2, 0.3, 0.4, and 0.5. Then, instead of identifying the optimal *p*-value threshold supervised by the phenotype *k*, we adopt the principal component GRS method (Coombes et al., 2020) and conduct a principal component analysis (PCA) on the 13 standardized GRSs, each obtained from a *p*-value threshold and is standardized to zero mean and unit standard deviation. For subject *i*, the resulting first principal component(s) (PC) score is used as the “final” GRS of phenotype *k*, and is denoted as *PCA*_*GRS*_*k*_ and referred to as PCA-GRS for phenotype *k*. In PCA-GRS, each SNP is reweighted so to maximize to GRS variation across all 13 *p*-values, and these weights are used to compute the PCA-GRS for phenotype *k* in the testing UPitt sample.

### 4.3. MetaGRS construction using elastic-net regression

Meta-GRS for AD is a weighted sum of individual GRSs for various phenotypes (Abraham et al., 2019). Because phenotypes could be correlated with each other, a composite GRS based on simple summation of the 25 PCA-GRS may conflate the effects. Similar to Abraham et al. (2019), we perform the elastic-net logistic regression in NACC using R package “glmnet” to determine the weights for each PCA-GRS for computing the MetaGRS. The model regresses AD status on the 25 standardized PCA-GRSs, adjusting for sex and the first five principal components for population stratification. The coefficients from the elastic-net regression indicate the contribution of each PCA-GRS to the risk of AD after capturing the genetic correlation between each phenotype. We trained and assessed the parameters in the elastic-net model with 20-fold cross-validations, and the parameters leading to the highest AUC were chosen for the final model. From the final model, the regression coefficients of the 25 PCA-GRSs are then used as the weights to compute MetaGRS for AD in the UPitt testing cohort, using the following formula:


(1) $$M\,e\,t\,a\,G\,R\,S=\sum_{k=1}^{25}\gamma_{k}\,P\,C\,A\,\_\,G\,R\,S_{k}^{s},$$

where $P\,C\,A\,\_\,G\,R\,S_{k}^{s}$ is the PCA-GRS for phenotype *k* standardized (denoted as *s*) to zero mean and unit standard deviation; γ_*k*_ is the weight associated with phenotype *k* obtained from the elastic-net regression.

### 4.4. Evaluation of predictive power of MetaGRS

We compute the Meta-GRS for each individual in the UPitt testing cohort by computing the weighted sum of the UPitt PCA-GRSs of different phenotypes with weightγ_*k*_ in Equation (1). We then evaluate the prediction performance of MetaGRS on AD status in comparison with a model that includes non-genetic covariates and AD PCA-GRS. We considered two predictive models for AD. The first model is to predict the age of onset of AD using Cox proportional hazard model, adjusting for age, sex, and first 5 principal components to capture the population stratification. The second model is to predict AD status using a logistic regression, adjusting for age, sex, and first 5 principal components. To evaluate the utility of MetaGRS, we compare the effect sizes in terms of hazard ratio in the Cox model and odds ratio in the logistic model. We also evaluate the predictive performance based on the C-index in the Cox model and pseudo-R^2^ and AUC in the logistic model.

## Data availability statement

The datasets presented in this study can be found in online repositories. The names of the repository/repositories and accession number(s) can be found in the article/Supplementary material.

## Author contributions

Study designed by KC, WF, C-LL, W-PL, S-YC, L-SW, and J-YT, with input from P-CH and HW. Analysis was completed by KC, WF, and C-LL. Manuscript written by KC, WF, and P-CH. All authors reviewed, edited, and approved the submitted version.
