# Supplementary figures and images for: The prediction of Alzheimer’s disease through multi-trait genetic modeling
Source: Front Aging Neurosci. 2023 Jul 27;15:1168638. doi: 10.3389/fnagi.2023.1168638 (PMC10416111; doi:10.3389/fnagi.2023.1168638)

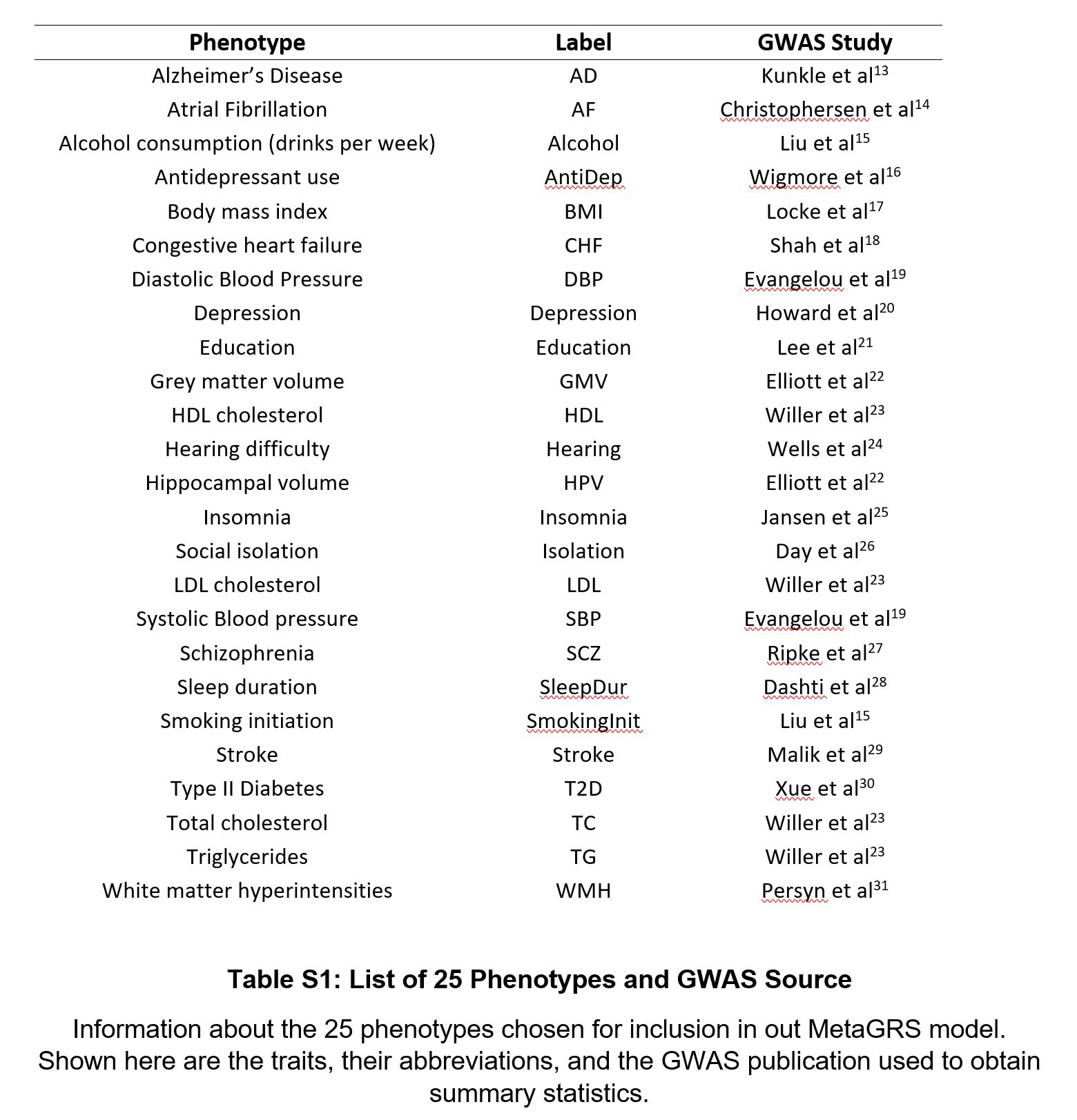

Supplement: Supplementary file 1 [file Image_1.jpeg]

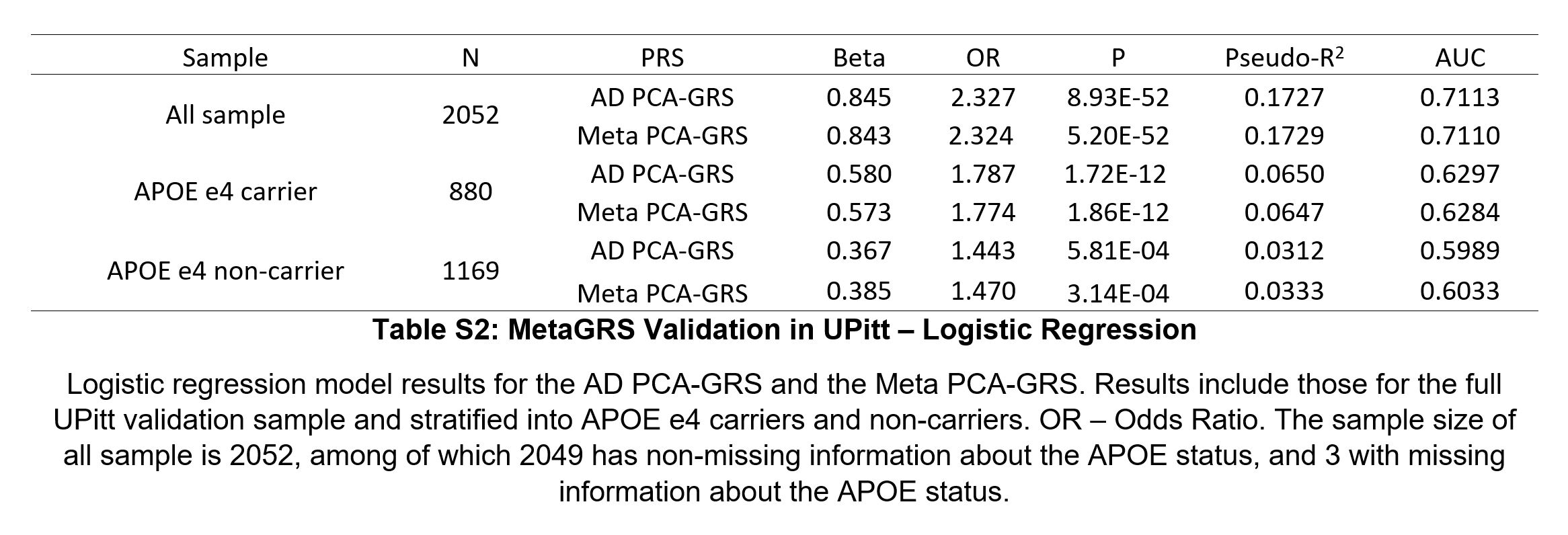

Supplement: Supplementary file 2 [file Image_2.jpeg]

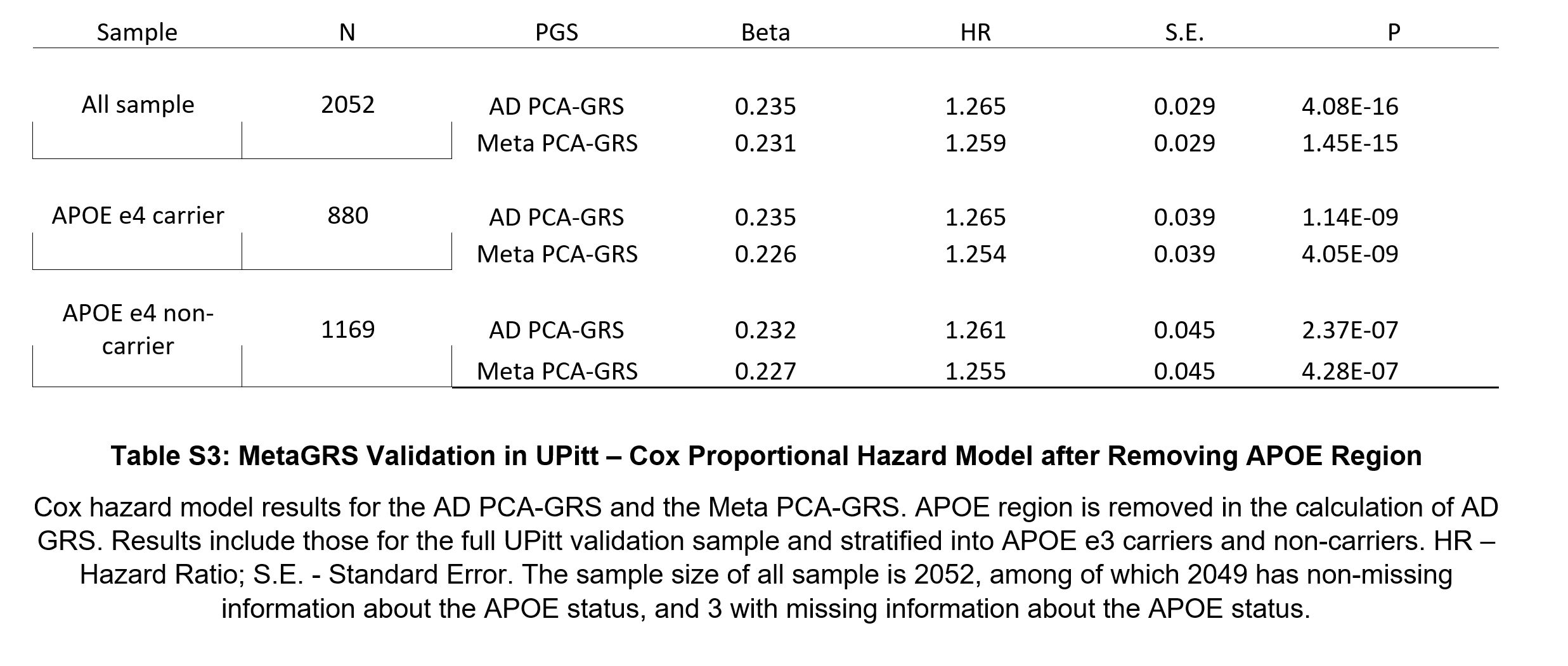

Supplement: Supplementary file 3 [file Image_3.jpeg]

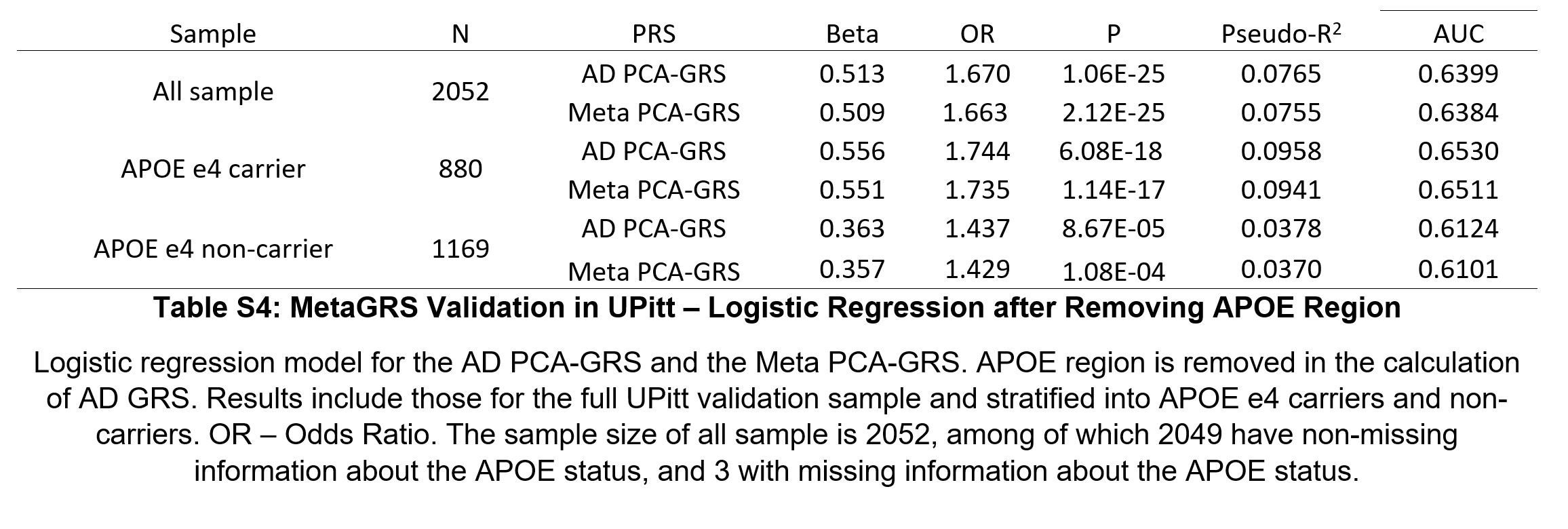

Supplement: Supplementary file 4 [file Image_4.jpg]

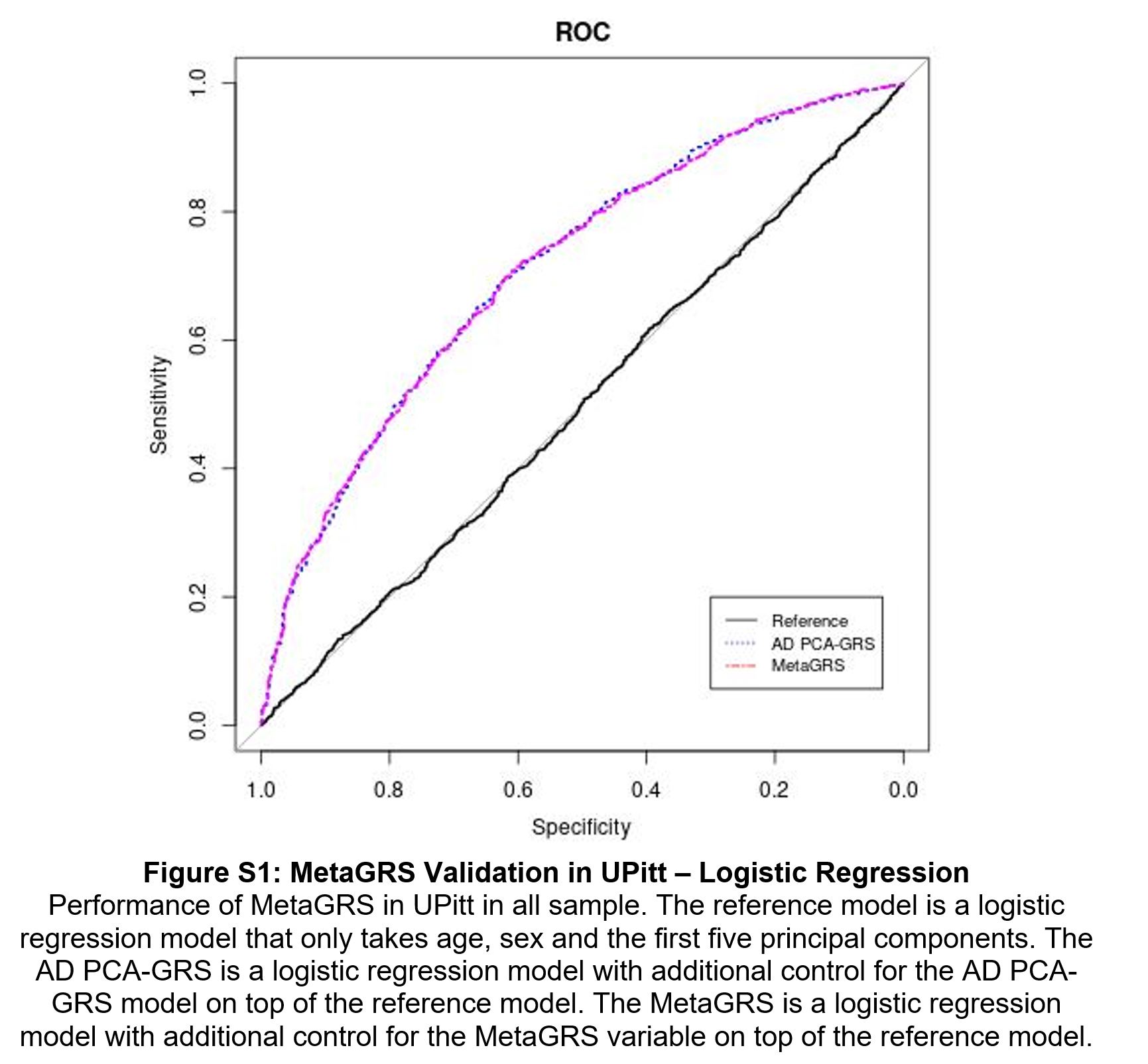

Supplement: Supplementary file 5 [file Image_5.jpg]

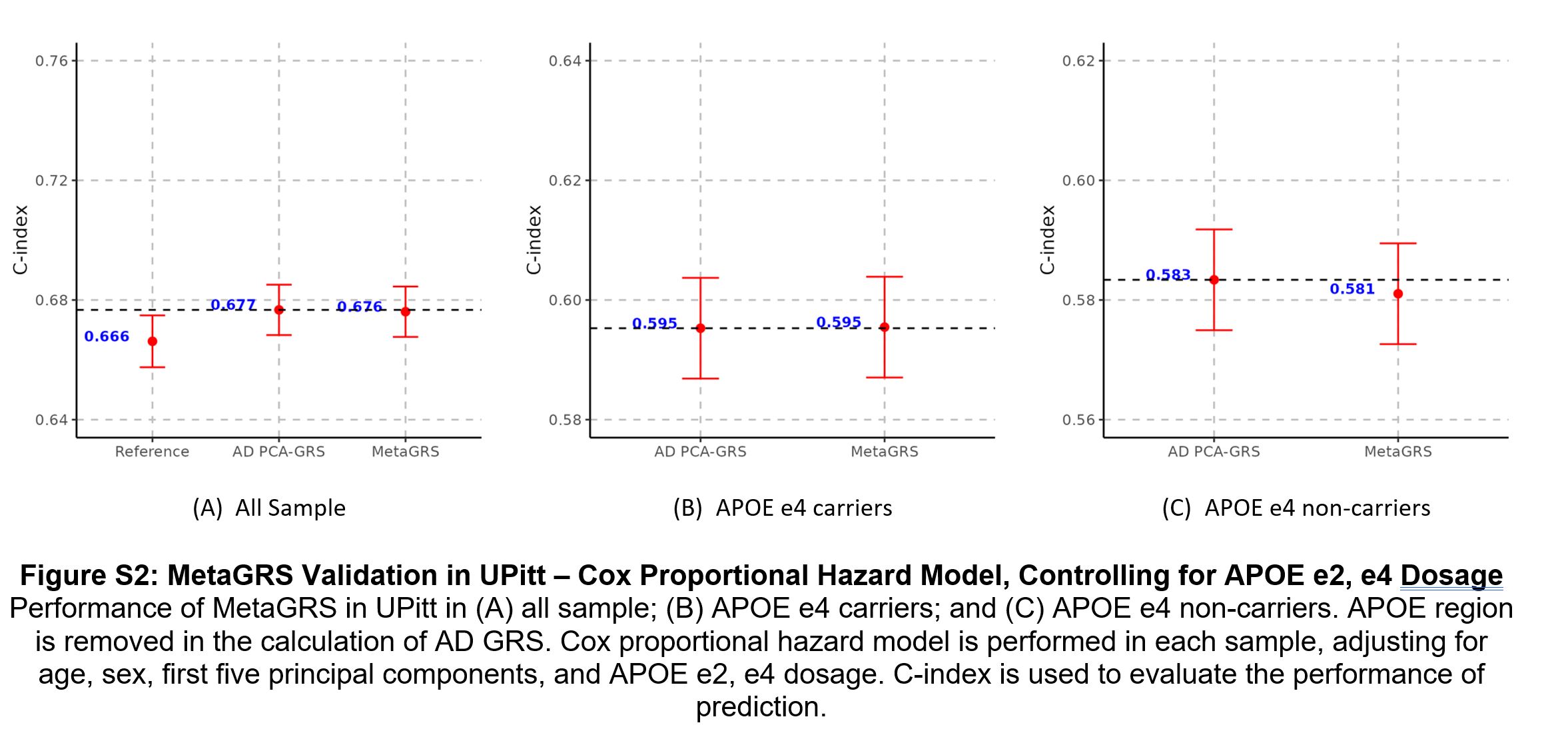

Supplement: Supplementary file 6 [file Image_6.jpg]

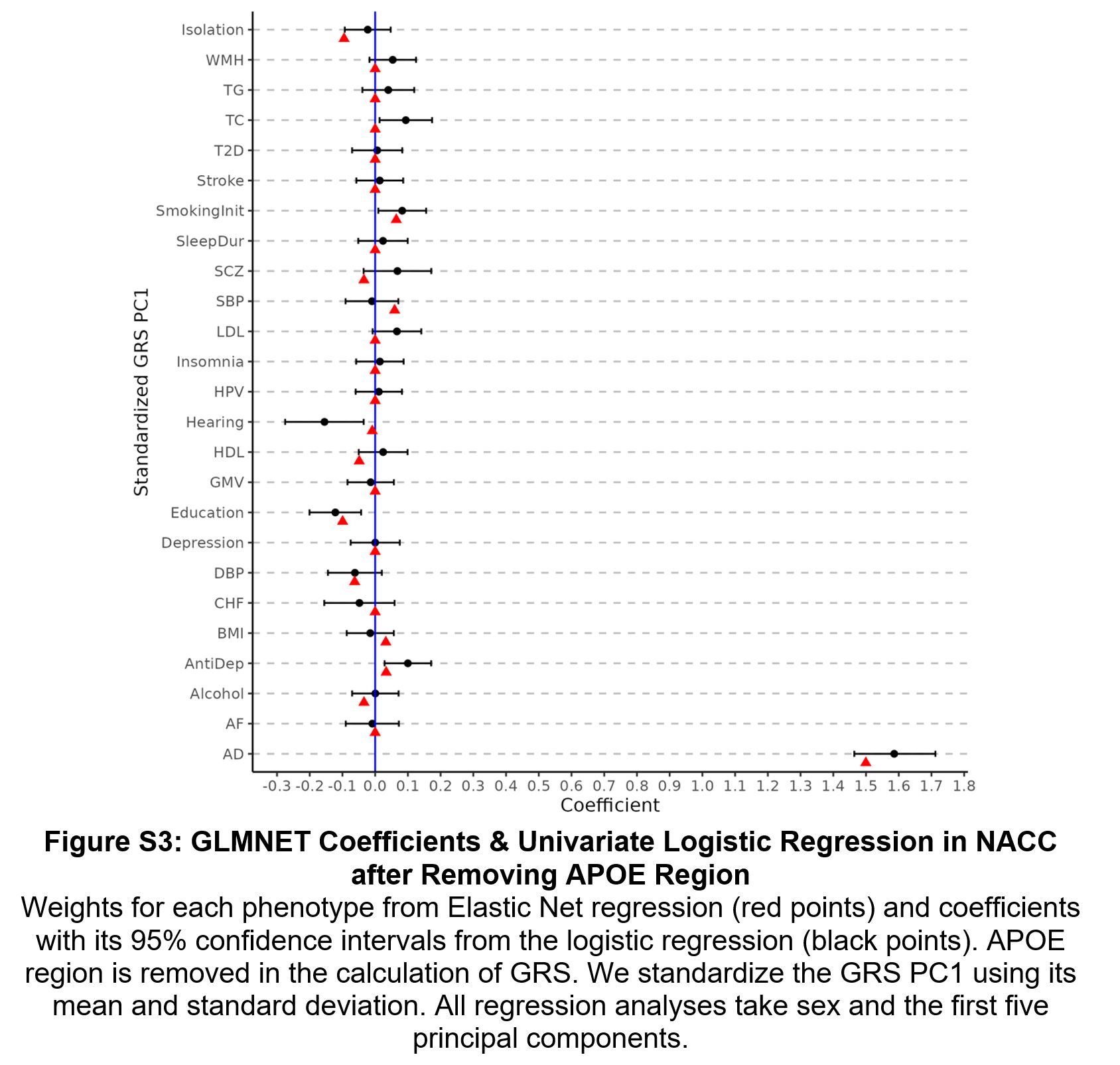

Supplement: Supplementary file 7 [file Image_7.jpg]
